# Investigation of Soluble and Transmembrane CTLA-4 Isoforms in Serum and Microvesicles

**DOI:** 10.4049/jimmunol.1303389

**Published:** 2014-06-13

**Authors:** Laura Esposito, Kara M. D. Hunter, Jan Clark, Daniel B. Rainbow, Helen Stevens, Jennifer Denesha, Simon Duley, Sarah Dawson, Gillian Coleman, Sarah Nutland, Gwynneth L. Bell, Carla Moran, Marcin Pekalski, John A. Todd, Linda S. Wicker

**Affiliations:** * Juvenile Diabetes Research Foundation/Wellcome Trust Diabetes and Inflammation Laboratory, Department of Medical Genetics, Cambridge Institute for Medical Research, University of Cambridge, Cambridge CB2 0XY, United Kingdom;; † National Institute for Health Research Cambridge Biomedical Research Centre, Addenbrooke’s Hospital, Cambridge CB2 0QQ, United Kingdom; and; ‡ Institute of Metabolic Science, University of Cambridge, Addenbrooke’s Hospital, Cambridge, CB2 0QQ, United Kingdom

## Abstract

Expression of the CTLA-4 gene is absolutely required for immune homeostasis, but aspects of its molecular nature remain undefined. In particular, the characterization of the soluble CTLA-4 (sCTLA-4) protein isoform generated by an alternatively spliced mRNA of *CTLA4* lacking transmembrane-encoding exon 3 has been hindered by the difficulty in distinguishing it from the transmembrane isoform of CTLA-4, Tm-CTLA-4. In the current study, sCTLA-4 has been analyzed using novel mAbs and polyclonal Abs specific for its unique C-terminal amino acid sequence. We demonstrate that the sCTLA-4 protein is secreted at low levels following the activation of primary human CD4^+^ T cells and is increased only rarely in the serum of autoimmune patients. Unexpectedly, during our studies aimed to define the kinetics of sCTLA-4 produced by activated human CD4^+^ T cells, we discovered that Tm-CTLA-4 is associated with microvesicles produced by the activated cells. The functional roles of sCTLA-4 and microvesicle-associated Tm-CTLA-4 warrant further investigation, especially as they relate to the multiple mechanisms of action described for the more commonly studied cell-associated Tm-CTLA-4.

## Introduction

The transmembrane isoform of CTLA-4 (Tm-CTLA-4) receptor plays a crucial role in the downregulation of the immune response and the maintenance of immune homeostasis, as shown by the lymphoproliferative syndrome and early lethality of CTLA-4–deficient mice (1–3). Tm-CTLA-4 is expressed by activated T cells, whereas it is constitutively expressed and required for regulatory T cell (Treg) suppression (4–6). At the molecular level, previous studies have provided evidence that an alternatively spliced mRNA of the CTLA-4 gene that lacks exon 3 is expressed in human, mouse, and rat immune cells (7, 8). As a result of splicing between exons 2 and 4, the predicted soluble CTLA-4 (sCTLA-4) isoform does not have a transmembrane domain or the membrane-proximal cysteine residue required for covalent homodimerization of the conventional Tm-CTLA-4 (9), thereby predicting a secreted, or soluble, isoform of monomeric CTLA-4 (sCTLA-4). The skipping of exon 3 predicts a shift in the reading frame, generating a C-terminal amino acid sequence that distinguishes sCTLA-4 from Tm-CTLA-4 (7). In both human and mouse sCTLA-4, mRNA expression is mainly detected in resting T cells, and its level is similar to that of Tm-CTLA-4 mRNA, whereas, following T cell activation, Tm-CTLA-4 is rapidly upregulated and becomes the predominant transcript (7, 8, 10–12). In humans, single nucleotide polymorphism (SNP) CT60 (rs3087243) in the 3′ untranslated region of human *CTLA4* is associated with multiple autoimmune diseases, including type 1 diabetes (T1D), Graves’ disease (GD), rheumatoid arthritis, and celiac disease (10, 13–16). At the cellular level, SNP CT60 is correlated with changes in mRNA levels of sCTLA-4; lower levels of sCTLA-4 mRNA were detected in resting CD4^+^ T cells and CD4^+^ CD25^+^ FOXP3^+^ Tregs of healthy donors carrying a T1D-susceptible genotype at SNP CT60 as compared with donors having the protective genotype (10, 17).

The extracellular domain of sCTLA-4, similar to that of the integral membrane isoform, contains the MYPPY motif involved in binding to the CD28-shared CD80/CD86 ligands on APCs. In a mixed lymphocyte response, recombinant sCTLA-4 showed immunomodulatory properties capable of suppressing cell proliferation in a dose-dependent manner (7). Levels ranging from 2 to 96 ng/ml material reported to be sCTLA-4 have been detected in the serum of patients with autoimmune thyroid diseases (18), systemic lupus erythematosus (19, 20), spondylarthropathies (20), celiac disease (21), Crohn’s disease (22), cutaneous systemic sclerosis (23), and T1D (24, 25) and were correlated with disease activity and clinical features (20–23). All of the studies on patients’ sera used Ig-based binding assays recognizing the extracellular domain of CTLA-4, not Abs specific for the soluble isoform of CTLA-4. The true molecular nature of the material in these sera recognized by anti–CTLA-4 Abs has been questioned (26) by the same laboratory that originally reported the increase of sCTLA-4 in autoimmune disease (18). Analysis of proteins immunoprecipitated from plasma donated by patients with autoimmune disease with a pool of anti–CTLA-4 Abs specific for the N-terminal CD80/CD86 binding domain of CTLA-4 has shown that the isolated molecules exhibited characteristics common to Igs and were able to interact with CD80 and CD86 ligands, but did not have the sequence of an isoform of CTLA-4 (26).

The accurate detection of human sCTLA-4 protein has been hampered by the lack of validated Abs that specifically target this isoform with high affinity. In this study, Abs that specifically recognize the recombinant soluble isoform of CTLA-4 have been generated and characterized to determine whether primary human T cells produce the sCTLA-4 protein in addition to expressing the alternatively spliced message and to evaluate sCTLA-4 levels in patients with autoimmune disease. We report that sCTLA-4 is secreted by in vitro activated human CD4^+^ T cells. However, sCTLA-4 is only rarely detected in serum samples from patients with autoimmune diseases or from healthy volunteers consistent with the findings of Oaks and colleagues (26). In addition to characterizing sCTLA-4 protein secreted from in vitro activated T cells, we observed that these cells also released Tm-CTLA-4 associated with nano-sized microvesicles (microvesicle CTLA-4 [mvCTLA-4]).

## Materials and Methods

### Subjects

Blood samples for CD4^+^ T cell purification were obtained from Cambridge BioResource donors not having any known autoimmune disease with the prior approval of the National Health Service Cambridgeshire Research Ethics Committee. Serum/Plasma collections were obtained from patients with T1D and GD. The 15 female and 3 male GD patients whose sera were tested ranged in age from 20 to 54 y; sera were collected prior to treatment. Sera from GD patients and from healthy donors (*n* = 15) matched in age and sex to the GD patients were provided by K. Chatterjee (University of Cambridge). T1D patients were 7 females and 7 males and ranged in age from 23 to 53 y. Ten T1D patients had comorbid medical conditions: 8 patients had GD, 1 had multiple sclerosis and GD, and 1 was affected with rheumatoid arthritis. Sera from healthy volunteers (*n* = 14) that were matched in age and sex with the T1D patients were analyzed as controls.

### RNA extraction and cDNA synthesis

Total RNA from purified human CD4^+^ T cells and splenic CD4^+^ T cells from C57BL/10 mice was extracted using TRIzol reagent (Invitrogen), according to the manufacturer’s instructions. First-strand cDNA from total RNA was synthesized using Superscript II reverse transcriptase (Invitrogen), according to the manufacturer’s instructions.

### Construction of recombinant expression vectors

The entire open reading frame encoding human CTLA-4 was obtained by PCR amplification of cDNA from human CD4^+^ T cells using the following primers: forward, 5′-GAACACCGCTCCCATAAAG-3′; reverse, 5′-GGTTTCTCAATTGATGGGAATAA-3′. The entire mouse *Ctla4* open reading frame was amplified by PCR of cDNA derived from CD4^+^ T cells isolated from C57BL/10 mice using the following primers: forward, 5′-GCCATGGCTTGTCTTGGACT-3′; reverse, 5′-AACGGCCTTTCAGTTGATG-3′. Human and mouse Tm-CTLA-4 and sCTLA-4 PCR products were gel purified with QIAquick Gel Extraction kit (QIAGEN) and cloned into the pcDNA3.1/V5-HIS-TOPO expression vector (Invitrogen), according to the manufacturer’s instructions. The *CTLA4* stop codon was included in the vector, thereby preventing the inclusion of the polyhistidine tag in the recombinant protein. Clones were verified by sequencing on an ABI PRISM 3700 DNA sequencer (Applied Biosystems). Endofree plasmid maxi kit (Qiagen) was used to generate endotoxin-free DNA for transfection.

### Generation of sCTLA-4–specific polyclonal Abs and mAbs

Abs reactive with sCTLA-4 but not Tm-CTLA-4 were generated in collaboration with Cambridge Research Biochemical (Cambridge, U.K.). Polyclonal Ab 4017 was generated by immunization of rabbits with a modified human sCTLA-4 synthetic peptide, C-Ahx-ENAPNRARM-acid, conjugated to keyhole limpet hemocyanin (KLH) through the N-terminal cysteine using *m*-maleimidobenzoyI-*N*-hydroxysuccinimide ester. Aminohexanoic acid was added to the sequence as a spacer to increase the distance of the peptide from the KLH protein carrier. Abs with reactivity against the sCTLA-4 peptide were affinity purified on a peptide-conjugated column, followed by depletion of any remaining anti-KLH Abs using a KLH-coupled affinity matrix. The 4B8 and 10D1 mAbs were developed following immunization of BALB/c mice with the modified sCTLA-4 synthetic peptide [C]-IAKEKKPSYNRGLSENAPNRARM-acid conjugated to KLH through the N-terminal cysteine. Spleen cells of mice with serum reactivity against both the synthetic peptide and recombinant sCTLA-4 were used for the production of hybridomas. sCTLA-4–specific Ab-producing clones 4B8 and 10D1 were identified by binding to the peptide used for immunization and verified by binding to recombinant human sCTLA-4 but not Tm-CTLA-4. Following repeated cloning to obtain stable production, bulk quantities of 4B8 and 10D1 (fermentation/purification) were obtained from Bio X Cell (West Lebanon, NH).

### HeLa transient cell transfection, total protein extracts, and cell supernatant collection

HeLa cells were grown in DMEM supplemented with 10% heat-inactivated FBS, 100 U penicillin and 100 μg streptomycin (Invitrogen), and 2 mM L-glutamine (Sigma-Aldrich) at 37°C in a humidified 5% CO_2_ incubator. For transient plasmid transfection of HeLa cells, PolyJet transfection reagent (SignaGen Laboratories) was used, according to the manufacturer’s protocol. HeLa cells were seeded at 7 × 10^5^ cells/well in a 6-well plate in complete DMEM and allowed to attach for 24 h before transfection. Transfected cells were harvested after 72 h with 0.25% trypsin–EDTA solution (Sigma-Aldrich). For tunicamycin treatment, transfected cells were cultured for 48 h and then incubated with 5 μg/ml tunicamycin for an additional 5 h. Washed cell pellets were resuspended in lysis buffer (50 mM Tris [pH 8.0], 150 mM NaCl, 2 mM EDTA, 1% IGEPAL CA-630 [Sigma-Aldrich]) containing protease and phosphatase inhibitors (1 mM NaF, 1 mM Na_3_VO_4_, 1% protease inhibitor mixture [Sigma-Aldrich]) for 30 min on ice, followed by centrifugation at 13,000 × *g* to remove cell debris. Culture supernatants from transfected HeLa cells were centrifuged at 500 × *g*, followed by filtration through a 0.22-μM filter. Cell lysates and culture supernatants were stored at −80°C.

### CD4^+^ T cell isolation and stimulation

CD4^+^ T cells were enriched by negative selection from whole blood freshly collected in heparinized tubes using an immunorosetting method, according to the manufacturer’s protocol (RosetteSep Human CD4^+^ T Cell Enrichment Cocktail; STEMCELL Technologies). CD4^+^ T cells were resuspended at 1–3 × 10^6^ cells/ml in exosome-depleted 10% FBS X-VIVO medium and stimulated either with plate-coated anti-CD3 (OKT3, 0.5 μg/ml; Bio X Cell, West Lebanon, NH) and anti-CD2 mAbs (T1.11, T1.12 1 μg/ml each; Abs-online.com) or with anti-CD3/CD28 Dynabeads (Invitrogen, Life Technologies) at a cell to bead ratio 1:0.5. Exosome-depleted medium was generated by overnight centrifugation at 100,000 × *g* at 4°C of X-VIVO medium plus 20% FBS. Medium was filtered through a 0.22-μm filter and diluted at a final concentration of 10% FBS with X-VIVO medium plus penicillin/streptomycin. Total cell lysates were made as described for HeLa cell transfectants and stored at −80°C.

### Western blot analysis

Samples were denatured at 70°C in NuPAGE LDS sample buffer (Invitrogen) for 10 min and separated on NuPAGE Novex 10% Bis-Tris or 3–8% Tris Acetate gels (Invitrogen) under reducing and nonreducing conditons. Lysates from 2 × 10^4^ and 5–10 × 10^3^ cells of sCTLA-4 and Tm-CTLA-4 HeLa transfectants or from 5–10 × 10^4^ anti-CD3/CD2–stimulated CD4^+^ T cells were loaded per well. Proteins were electrophoretically transferred to Invitrolon polyvinylidene difluoride (Invitrogen) membranes in transfer buffer (2.5 mM Tris, 19.2 mM glycine, 0.1% SDS) with 20% methanol. Membranes were blocked with 2% ECL Advance Blocking Agent (GE Healthcare) in PBS with 0.05% (w/v) Tween 20 (PBS-T). Primary Abs against specific proteins were incubated in 1% ECL Advance Blocking Agent PBS-T overnight at 4°C. Ab reactivity was detected following 1-h incubation at room temperature with peroxidase-conjugated secondary Abs using a chemiluminescent detection system (Pierce ECL 2 Western blotting substrate; Thermo Scientific) and visualized by exposure to Amersham Hyperfilm ECL (GE Healthcare). Primary and secondary Abs used were as follows: anti–CTLA-4 (C19), a polyclonal Ab generated in goat using the C-terminal sequence from Tm-CTLA-4 (Santa Cruz Biotechnology), followed by Pierce peroxidase-conjugated recombinant protein G (Thermo Scientific), anti-CD3ζ (D-4), anti-HLA class I (HP1F7) (Santa Cruz Biotechnology), anti-CD63 (H5C6), anti-CD107b (lysosomal-associated membrane protein 2 [LAMP2], H4B4) (BD Pharmingen), anti–sCTLA-4 4B8 and anti–sCTLA-4 10D1, followed by peroxidase-conjugated anti-mouse IgG (Santa Cruz Biotechnology), and anti–sCTLA-4 4017, followed by peroxidase-conjugated anti-rabbit IgG (Vector Laboratories).

### Immunoprecipitation

Immunoprecipitation was performed using Dynabeads Coimmunoprecipitation Kit (Invitrogen), according to manufacturer’s instructions. Briefly, 6 μg 4B8 or isotype control mouse plus IgG2b mAb (BioLegend) was coupled to 5 mg Dynabeads M-270 Epoxy (Life Technologies). Between 1.5 and 2 mg coupled Dynabeads were used to immunoprecipitate sCTLA-4. Immunoprecipitation was carried out overnight at 4°C under rotation, followed by elution of precipitated protein in the provided extraction buffer (pH 2.8).

### Isolation of microvesicles

Microvesicle isolation was performed on supernatant harvested from CD4^+^ T cells cultured in X-VIVO/10% FBS exosome-depleted medium after 72-h stimulation with plate-coated anti-CD3 and anti-CD2. Microvesicle isolation was based on the protocol previously published (27). CD4^+^ T cell culture supernatants were sequentially centrifuged to eliminate cell debris, apoptotic bodies, and microvesicles >100 nm (all carried out at 4°C), as follows: 300 × *g* for 5 min, 1,200 × *g* for 20 min, and 10,000 × *g* for 30 min (28, 29). Final supernatants were filtered through a 0.22-μM filter and ultracentrifuged at 100,000 × *g* for 1 h at 4°C; pellets were resuspended in 10 ml ice-cold PBS and ultracentrifuged at 100,000 × *g* for 1 h. Microvesicle isolation from plasma and serum samples was performed, as previously published (29). In brief, 5–7 ml plasma or serum samples diluted 1:1 in Dulbecco’s PBS (GE Healthcare) were centrifuged at 2,000 × *g* for 30 min and 12,000 × *g* for 45 min, followed by 2 h at 110,000 × *g*. Pellets were resuspended in 10 ml ice-cold PBS, filtered through a 0.22-μM filter, and ultracentrifuged at 110,000 × *g* for 70 min; the 10-ml wash and ultracentrifugation steps were repeated. Microvesicle-containing pellets were resuspended in 100 μl PBS for electron microscopy and Western blot analyses or in 100 μl of the 1% IGEPAL lysis buffer described earlier for testing in the CTLA-4 immunoassays.

### Immunoelectron microscopy

Microvesicle fractions resuspended in PBS obtained from ultracentrifugation of culture supernatants of CD4^+^ T cells after 72 h of stimulation were bound to glow-discharged formvar-coated grids contrasted with 1% uranyl acetate. Immunogold labeling of vesicles for CD63 was performed by staining with mouse anti-human CD63 mAb MEM-259 (Abcam), followed by goat anti-mouse IgG coupled to 10 nm gold (BB International). Samples were viewed with a FEI Tecnai G20 transmission electron microscope operated at 120 kV. Images were captured using an AMT XR60B (Deben) digital camera running Deben software.

### Flow cytometric analysis

HeLa cells were dissociated with trypsin, washed twice in PBS, fixed with Cytofix/Cytoperm (BD Biosciences) for 20 min at 4°C, washed twice with 1× Perm/Wash (BD Biosciences), and resuspended in 1× Perm/Wash. sCTLA-4–specific rabbit anti-human 4017 polyclonal Ab, mouse IgG1 anti-human 10D1 mAb, and mouse IgG2b anti-human 4B8 mAb were labeled with Zenon Alexafluor 647 rabbit IgG labeling kit, Zenon Alexafluor 647 mouse IgG1 labeling kit, or Zenon AlexaFluor 647 mouse IgG2b labeling kit (Molecular Probes, Life Technologies). Matched isotype controls, rabbit IgG (Southern Biotech), and mouse IgG1 and IgG2b (BioLegend) were similarly labeled and used as negative controls. Cells were stained with each of the above Abs together with anti-CD152/CTLA-4 PE BN13 or a mouse IgG2a PE (BD Biosciences) isotype control for 30 min at 4°C, washed twice, and resuspended in Cell Wash (BD Biosciences). For microvesicle staining, 50–100 μl microvesicles purified from CD4^+^ T cell culture supernatant or plasma/serum were incubated with 10 μl 4 μM aldehyde/sulfate polystyrene latex beads (Molecular Probes, Invitrogen) for 15 min at room temperature. The volume was brought to 1 ml with PBS, followed by gentle rotation for 2 h. Any remaining binding sites on the beads were then saturated by incubation with glycine (100 mM final in PBS) for 30 min, washed three times with PBS/0.5% BSA, and resupended in 500 μl PBS/BSA. A total of 10 μl coated beads was incubated for 30 min at 4°C with anti-CD63 PE (H5C6) or anti-CD152 PE (BN13) or their respective isotype controls, mouse IgG1 PE, or mouse IgG2a PE, and washed twice with PBS/BSA.

Samples were processed on a BD LSRFortessa cell analyzer (BD Bioscience). Data were analyzed with FlowJo software (Tree Star).

### Immunoassays for human CTLA-4 and sCTLA-4

#### Human pan–CTLA-4 immunoassay.

Ninety-six–well enzyme immunoassay/RIA flat-bottom plates (Corning Costar) were coated with 100 μl either anti-CD152 BN13 mAb or mouse IgG2aκ (clone G155-178; BD Pharmingen) isotype control diluted at 2 μg/ml in coating buffer (0.1 M sodium phosphate [pH 9]). After overnight incubation at 4°C, plates were washed with PBS-T (0.05% Tween 20 in PBS), and 100 μl sample diluted in DMEM/10% FBS/0.05% Tween 20 was added to each well (Tween 20 is added to prevent nonspecific binding throughout the assay following the coating step). Following overnight incubation at 4°C and plate washing with PBS-T, detection was performed by addition of 100 μl/well anti–CTLA-4 biotinylated mAb clone 14D3 (eBioscience) at 2 μg/ml in PBS-T for 1 h at 4°C. Wells were washed with PBS-T, and the bound biotinylated protein was detected by incubation with 100 μl/well 0.1 μl/ml europium-labeled streptavidin (PerkinElmer) for 1 h at 4°C, followed by washes in PBS-T and addition of 100 μl/well DELFIA Enhancement Solution (PerkinElmer). Measurements were performed with a time-resolved Victor X reader (PerkinElmer). Each assay point was performed in duplicate. A standard curve was generated with the use of serial dilutions of a commercially available human CTLA-4/Fc fusion protein (Chimerigen Laboratories). sCTLA-4 concentrations in HeLa supernatants were estimated in this assay format considering a molar equivalent of one 90-kDa dimeric CTLA-4/Fc with two sCTLA-4 monomers each with a predicted molecular mass of 15.4 kDa.

#### Human sCTLA-4 immunoassay.

Ninety-six–well plates were coated with BN13 or isotype control, as described above, for the human CTLA-4 assay, and 180 μl sample diluted in DMEM/10% FBS/0.05% Tween 20 was added to each well. After overnight incubation at 4°C and without washing, 20 μl/well 0.1 μg/ml 4B8 diluted in DMEM/10% FBS/0.05% Tween 20 was added to each sample. Omitting the wash step that would normally be performed in most binding protocols increased the sensitivity of the assay ∼2-fold. Following overnight incubation at 4°C, wells were washed with PBS-T, and 100 μl/well rat anti-mouse IgG2b biotin mAb (clone RMG2b-1; BioLegend) at 200 ng/ml in PBS-T was added. The plate was incubated 1 h on ice. Detection of biotinylated protein was carried out as described for the human CTLA-4 assay.

### Statistical test

Comparisons of means between two groups were performed using the Mann–Whitney *U* test.

## Results

### Characterization of anti-human sCTLA-4 Abs

**FIGURE 1.**
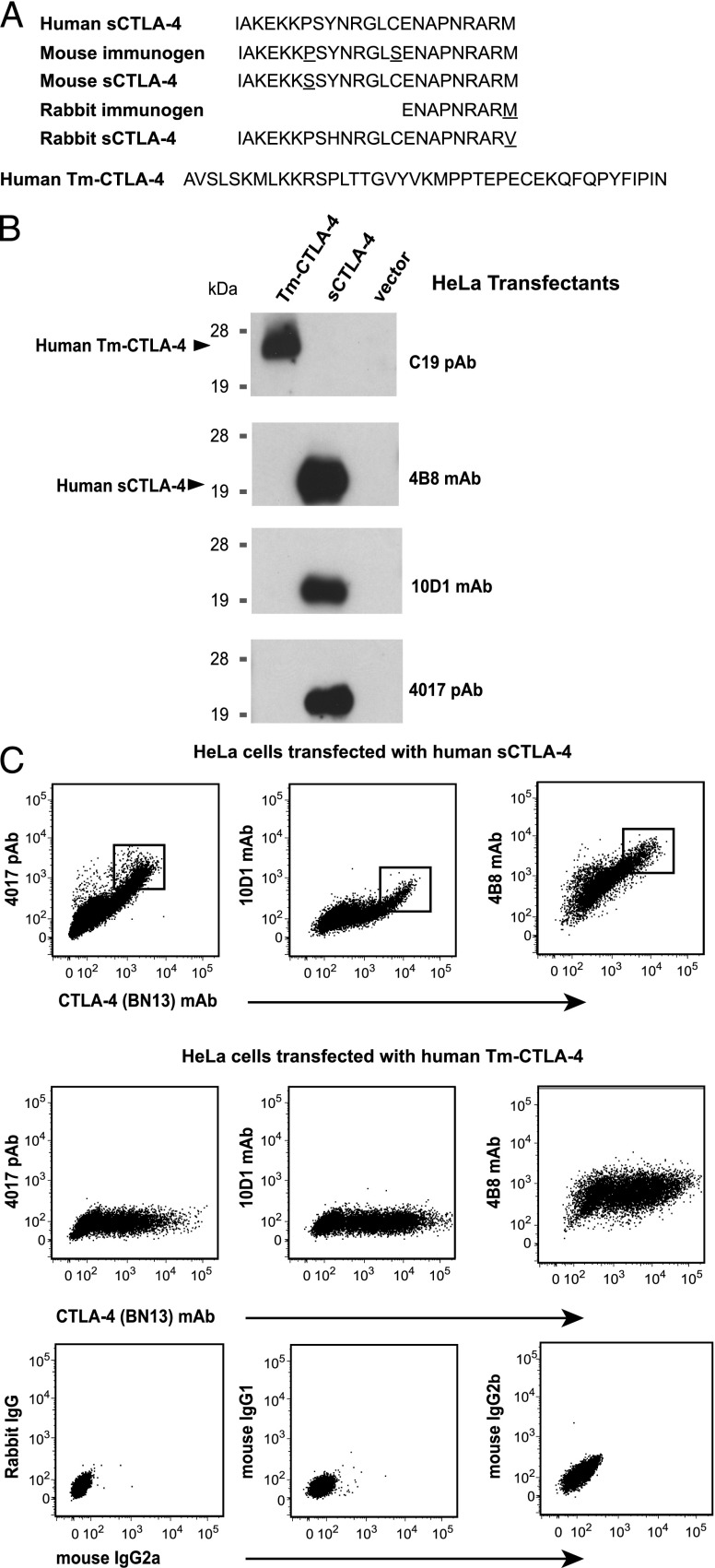
Development of Abs specific for the sCTLA-4 isoform. (**A**) Human (NP_001032720.1), rabbit, and mouse (ENSMUSP00000095327) amino acid sequences at the C-terminal region of sCTLA-4 are aligned with the synthetic peptides used for immunization of rabbits (P1_rabbit) and mice (P2_mouse). The cytoplasmic tail of TM-CTLA-4 is shown for comparison at the *bottom* of the panel (P16410, UniProt). Mouse and human nucleotide sequences encoding sCTLA-4 have been confirmed in our laboratory by sequencing. Rabbit amino acid sequence was determined from the deposited Tm-CTLA-4 nucleotide sequence (CTLA4_rabbit ENSOCUG00000017274) after alignment with human sCTLA-4 nucleotide sequence (NM_001037631.2). The 9-aa peptide used to immunize rabbits differs at a single residue from the rabbit sequence, a methionine for valine substitution at position 174 of rabbit sCTLA-4. The 23-aa synthetic peptide having the human sequence used to immunize mice differs at position 158 from the endogenous mouse sequence; a proline replaces serine. A serine for cysteine substitution in the 23-aa peptide was made at the residue corresponding to position 165 of the mature sCTLA-4 protein to prevent the formation of disulfide bonds in the peptide preparation and the peptide-KLH conjugate used for immunization. (**B**) The 4B8 mAb, 10D1 mAb, and 4017 polyclonal Ab validation by Western blot under reducing conditions on detergent lysates of HeLa cells expressing human recombinant Tm-CTLA-4, sCTLA-4, and vector only. Specific detection of Tm-CTLA-4 is performed with CTLA-4 (C19) polyclonal Ab. (**C**) sCTLA-4 Ab specificity was confirmed by flow cytometry using HeLa cells transfected with sCTLA-4 (*top row*) or Tm-CTLA-4 (*middle row*). As a control, HeLa cells transfected with Tm-CTLA-4 were stained with isotype-matched control Abs (*bottom row*); similar results were obtained using HeLa cells transfected with sCTLA-4 (data not shown). All experiments are representative of at least three independent observations.

A polyclonal Ab, termed 4017, was raised in rabbit against a synthetic peptide corresponding to the final nine C-terminal amino acid residues (ENAPNRARM) at position 166–174 of human sCTLA-4, and two mAbs, 10D1 and 4B8, were raised in mouse against the 23 aa unique to sCTLA-4 (Fig. 1A). Their specific binding to sCTLA-4 was validated by Western blot (Fig. 1B) and flow cytometric (Fig. 1C) analyses using HeLa cells expressing recombinant sCTLA-4 and Tm-CTLA-4 proteins. In both assays, all three Abs, 4017, 4B8, and 10D1, bound to human sCTLA-4, but not to human Tm-CTLA-4, which has a different amino acid sequence at the C-terminal region of the protein (Fig. 1A). Production of Tm-CTLA-4 by the transfected HeLa cells was confirmed using a commercially available polyclonal Ab (C19) generated by immunizing goats with the C-terminal amino acid sequence unique to the transmembrane isoform (Fig. 1B). As expected, C19 did not bind to recombinant human sCTLA-4. One clone, BN13, that recognizes an epitope in the CD80/CD86 binding domain common to sCTLA-4 and Tm-CTLA-4 can be used to detect the intracellular expression levels of both recombinant sCTLA-4 and Tm-CTLA-4 in transfected HeLa cells by flow cytometry (Fig. 1C). All three sCTLA-4–specific Abs costained along with BN13 the transfected HeLa cells expressing sCTLA-4, but not the Tm-CTLA-4 transfectants.

### The 10D1 mAb and 4017 polyclonal Ab specific for human sCTLA-4 bind mouse sCTLA-4

Human, rabbit, and mouse sCTLA-4 C-terminal regions share 95% amino acid sequence identity. However, rabbit and mouse sCTLA-4 molecules differ from the human sequence at distinct residues (Fig. 1A), raising the possibility that the anti-human sCTLA-4 Abs we have generated may in these two species recognize distinct epitopes on sCTLA-4. Data consistent with this hypothesis were obtained when we tested the two mAbs, 4B8 and 10D1, and the polyclonal Ab 4017 on mouse sCTLA-4 recombinant protein by Western blot (Supplemental Fig. 1). Because human and mouse sCTLA-4 molecules share a methionine at the final residue of sCTLA-4, but differ from the rabbit sequence at the final residue of sCTLA-4, it was not surprising that we observed binding to mouse sCTLA-4 recombinant protein by the rabbit polyclonal anti-human sCTLA-4 Abs (Supplemental Fig. 1A, *top panel*). The immunizing peptide for the rabbits contained only the final 9 aa of the sCTLA-4 protein; therefore, it is highly likely that the binding of the rabbit polyclonal Ab is focused on the terminal methionine residue. The expected lack of cross-reactivity to mouse sCTLA-4 of a mouse mAb generated by immunization with a peptide of human sCTLA-4 was observed with the 4B8 mAb (Supplemental Fig. 1A, *lower panel*), indicating that 4B8 binding most likely requires the human sequence-specific proline residue at position 158 instead of the mouse serine residue (Fig. 1A). Unexpectedly, the 10D1 anti-human sCTLA-4 mAb did bind to mouse sCTLA-4 (Supplemental Fig. 1A, *middle panel*), indicating that the human-specific proline residue is not required for binding by the 10D1 mAb and that the epitopes in the C-terminal region recognized by the two anti-human sCTLA-4 mAb are not identical. As predicted, no binding of anti-human sCTLA-4 Ab to recombinant mouse Tm-CTLA-4 was observed (Supplemental Fig. 1A, *right lanes*).

### Human and mouse sCTLA-4 isoforms are expressed as glycosylated monomers

**FIGURE 2.**
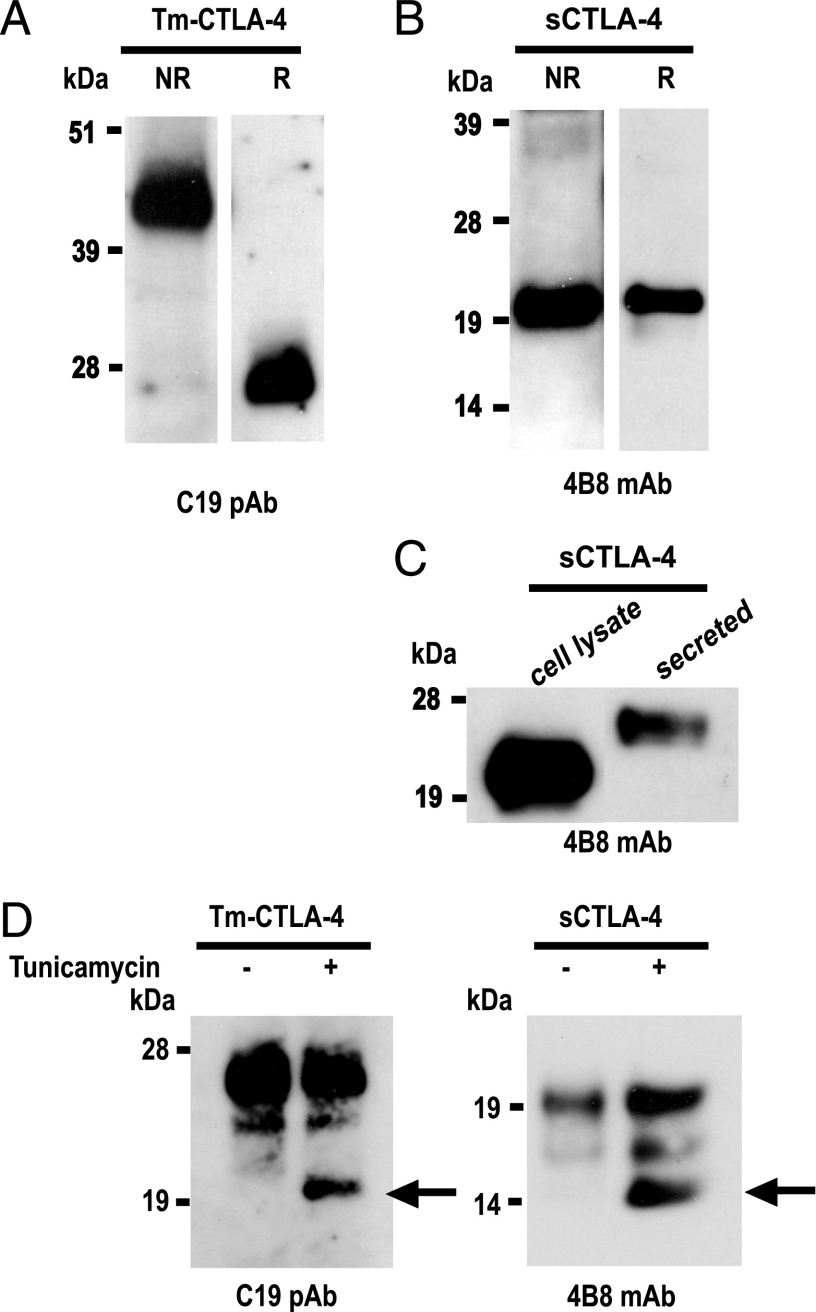
sCTLA-4 is secreted and expressed as a glycosylated monomer. (**A**) Human Tm-CTLA-4 protein is detected as a dimer under nonreducing conditions (NR) and as a monomer under reducing conditions (R) in lysates from HeLa cells transfected with human Tm-CTLA-4 cDNA. (**B**) Lysates from HeLa cells transfected with human sCTLA-4 cDNA contain sCTLA-4 protein that migrates as a monomer in both nonreducing and reducing conditions. (**C**) Under reducing conditions, sCTLA-4 protein secreted into the culture medium of HeLa cells transfected with sCTLA-4 cDNA migrates more slowly than sCTLA-4 present in cell lysates. (**D**) N-glycan addition contributes to the molecular masses of Tm-CTLA-4 and sCTLA-4. Following culture with or without tunicamycin, detergent lysates from HeLa cells transfected with Tm-CTLA-4 or sCTLA were analyzed by Western blot under reducing conditions using C19 polyclonal Ab or 4B8 mAb, respectively. Arrows indicate the protein species lacking N-linked glycans. All experiments are representative of at least three independent observations.

Tm-CTLA-4 covalent homodimerization, required for its high-avidity binding to CD80/CD86 ligands, is mediated by a cysteine at position 122 (9). As expected, under nonreducing conditions, recombinant Tm-CTLA-4 present in detergent lysates of transfected HeLa cells migrated as a dimer of ∼42–45 kDa and under reducing conditions as a monomer of ∼26–28 kDa (Fig. 2A). The soluble isoform, as a consequence of exon 3 skipping, lacks this cysteine residue, predicting that the protein is expressed as a monomer. Indeed, Western blot analysis under both nonreducing and reducing conditions of detergent lysates from HeLa cells transfected with human sCTLA-4 showed a single immunoreactive species corresponding to the monomeric protein with an apparent molecular mass of ∼20–22 kDa (Fig. 2B). Furthermore, sCTLA-4 was present in HeLa cell culture medium, although with a higher molecular mass (∼24–26 kDa) than that observed for the cell-associated protein, suggesting that, once the sCTLA-4 is fully glycosylated, it is secreted by the transfected cells (Fig. 2C). Human Tm-CTLA-4 and sCTLA-4 monomers have predicted molecular masses based on their unmodified full-length amino acid sequences of 20.6 and 15.4 kDa, respectively, which are lower than the apparent molecular mass observed by Western blot analysis. The extracellular regions of both Tm-CTLA-4 and sCTLA-4 contain, at positions 113 and 145, two potential sites for *N*-linked glycosylation. To investigate whether the observed shift in molecular mass is the consequence of posttranslational modification, HeLa transfectants expressing sCTLA-4 and Tm-CTLA-4 recombinant proteins were incubated with tunicamycin, an inhibitor of N-glycosylation, followed by Western blot analysis of cell lysates. Consistent with the presence of *N*-linked glycosylation sites on both CTLA-4 isoforms, the addition of tunicamycin revealed novel immunoreactive species in the lysates from HeLa cells expressing either isoform (Fig. 2D). The migration positions of the tunicamycin-dependent protein bands for both isoforms that presumably lack the addition of any carbohydrate moieties were in agreement with their predicted apparent molecular masses (Fig. 2D).

As demonstrated by Western blot analysis and similar to its human counterparts, mouse sCTLA-4 migrates as a monomer and mouse Tm-CTLA-4 migrates as a dimer (Supplemental Fig. 1B). Detergent lysates from HeLa cells transfected with mouse sCTLA-4 contained immunoreactive species with an apparent molecular mass of ∼24–26 kDa (Supplemental Fig. 1B, 1C) and, similar to the observations made with cells expressing human sCTLA-4, mouse sCTLA-4 secreted from transfected HeLa cells migrated as a larger protein species of ∼28 kDa (Supplemental Fig. 1C). Mouse Tm-CTLA-4 is expressed as a dimer of ∼48–50 kDa consisting of two monomeric subunits of ∼28–30 kDa (Supplemental Fig. 1B). Mouse Tm-CTLA-4 and sCTLA-4 monomers have predicted molecular masses of 20.9 and 15.4 kDa, respectively. However, by Western blot analysis, we found that both mouse recombinant proteins migrated not only at a higher molecular mass than predicted from their amino acid sequence but also higher than that of the corresponding human isoforms (Supplemental Fig. 1C and compare Supplemental Fig. 1B, 1D with Fig. 2A, 2B, 2D). These apparent mass differences were postulated to be the consequence of increased posttranslational N-glycosylation for the mouse proteins as compared with the human isoforms. Indeed, tunicamycin treatment of HeLa cells transfected with mouse Tm-CTLA-4 and sCTLA-4 yielded products that migrated at the expected molecular masses (Supplemental Fig. 1D).

### In vitro activated human CD4^+^ T cells secrete sCTLA-4

To optimize our ability to quantify the production of sCTLA-4 from human cells, we tested a number of potential binding assay formats utilizing the mAbs specific for sCTLA-4 as capture and detection reagents together with various mAbs binding the sCTLA-4 isoform via its N-terminal CD80/CD86 binding domain. The most sensitive format of the assay identified uses N-terminal–specific anti–CTLA-4 mAb BN13 as the capture Ab and sCTLA-4–specific 4B8 mAb as the detection Ab; the lower limit of detection of recombinant sCTLA-4 with this assay is ∼2 pg/ml (Supplemental Fig. 2A, *right panel*). Recombinant sCTLA-4 secreted from transfected HeLa cells is used to generate the standard curve for quantifying the levels of sCTLA-4 in serum, cell culture supernatants, and cell lysates. The quantification of recombinant sCTLA-4 was derived from its activity in a pan–CTLA-4 assay based on two mAbs both recognizing the N-terminal region of CTLA-4, which is present in both sCTLA-4 and Tm-CTLA-4, using a commercially available fusion protein between CTLA-4 and the Fc portion of human IgG1 (CTLA-4/Fc) as a standard (Supplemental Fig. 2B). The pan-CTLA-4 assay, with a lower limit of sensitivity of ∼20 pg/ml, is 10-fold less sensitive than the sCTLA-4–specific assay in detecting sCTLA-4 (Supplemental Fig. 2C, *right panel*). Having validated the specificity of the sCTLA-4 Abs and developed a sensitive binding assay, we then investigated whether sCTLA-4 protein is produced by human CD4^+^ T cells.

**FIGURE 3.**
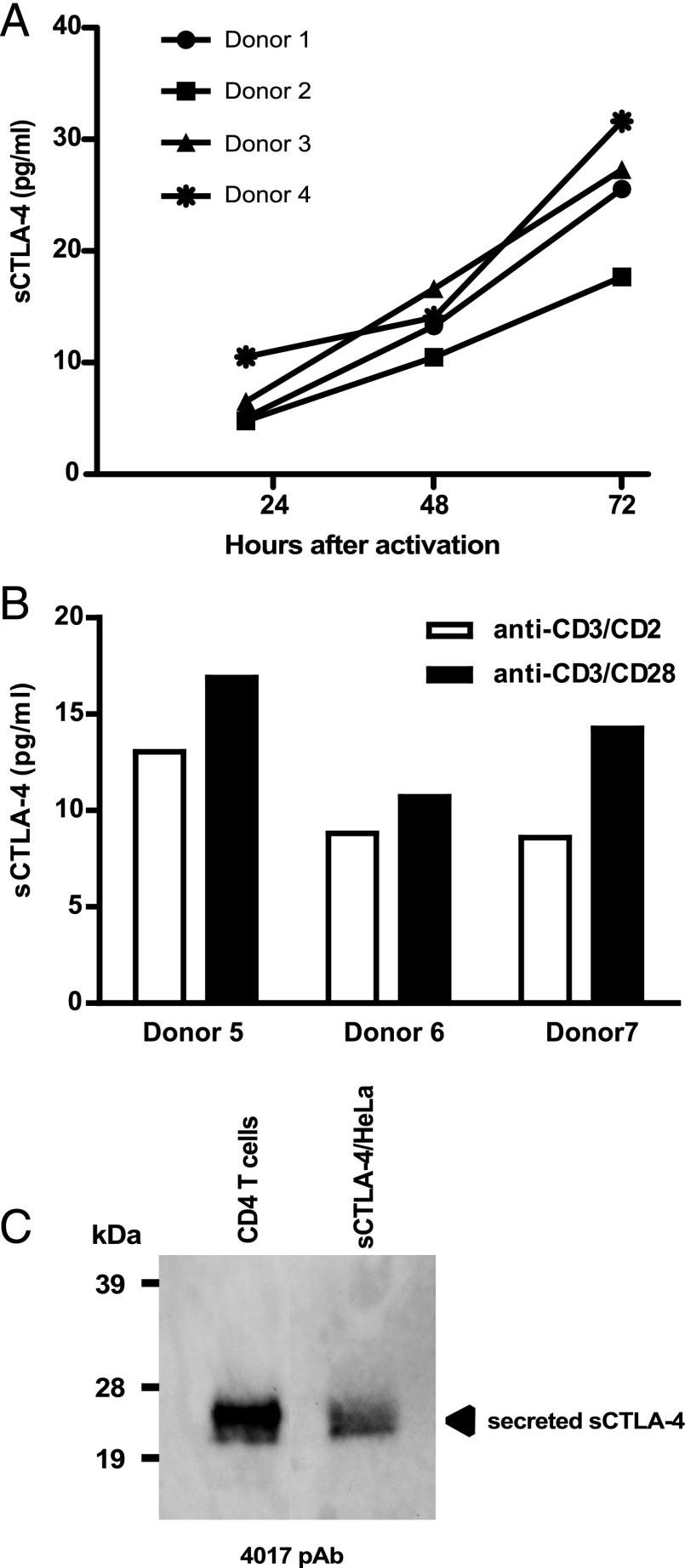
(**A**) In vitro activation of CD4^+^ T cells induces secretion of sCTLA-4. CD4^+^ T cells of four healthy donors were stimulated in vitro with anti-CD3/CD2 mAb and culture supernatants harvested at the indicated time points. CD4^+^ T cells (3 × 10^6^ cells/ml) from the four donors were cultured on separate days. Isotype controls were negative for all samples (data not shown). (**B**) Secretion of sCTLA-4 by CD4^+^ T cells (1 × 10^6^ cells/ml) of three independent donors stimulated for 72 h in vitro with either anti-CD3/CD2 mAb or anti-CD3/CD28 microbeads. (**C**) Detection of sCTLA-4 following immunoprecipitation and Western blot analysis. sCTLA-4 protein in culture supernatants from activated CD4^+^ T cells and from HeLa cells transfected with sCTLA-4 was immunoprecipitated using 4B8 mAb, followed by 4017 polyclonal Ab immunoblotting. No sCTLA-4 was immunoprecipitated with the corresponding IgG2b isotype control (data not shown). Western blot gel is representative of more than three independent experiments.

CD4^+^ T cells isolated from whole blood of four healthy donors were analyzed for sCTLA-4 and Tm-CTLA-4 proteins ex vivo and after 24, 48, and 72 h of activation with anti-CD3/CD2 Abs. Cell lysates and culture supernatants were assessed using the specific and highly sensitive sCTLA-4 mAb-based assay described above. sCTLA-4 was detected in CD4^+^ T cell supernatants after activation in a time-dependent manner ranging between 17 and 32 pg/ml after 72 h in culture (Fig. 3A). Similar levels of sCTLA-4 were detected in the supernatants of CD4^+^ T cells purified from three healthy donors comparing two activation protocols, as follows: anti-CD3/CD28 microbeads and anti-CD3/CD2 mAbs (Fig. 3B). Probably as a consequence of the low expression levels and rapid secretion of the mature protein, we did not detect sCTLA-4 in detergent lysates from CD4^+^ T cells before or after activation by immunoassay or Western blot analysis. Similarly, sCTLA-4 was not detected in activated CD4^+^ T cells by flow cytometry using any of the three sCTLA-4–specific Abs (data not shown).

sCTLA-4 protein in culture supernatants from activated CD4^+^ T cells detected by immunoassay could not be directly detected by Western blot analysis as a result of the detection limit of ∼240 pg/ml, compared with the 2 pg/ml detection limit of the sCTLA-4 immunoassay (Supplemental Fig. 2A, 2D). Therefore, to provide further evidence that sCTLA-4 is secreted by CD4^+^ T cells, immunoprecipitation with anti–sCTLA-4 mAb followed by Western blot analysis was performed using culture supernatants from T cells activated for 72 h (Fig. 3C). Importantly, T cell–derived sCTLA-4 migrated equivalently to recombinant sCTLA-4 secreted from transfected HeLa cells, indicating that sCTLA-4 secreted by primary T cells is glycosylated.

### Release of Tm-CTLA-4 following CD4^+^ T cell activation is associated with microvesicles

**FIGURE 4.**
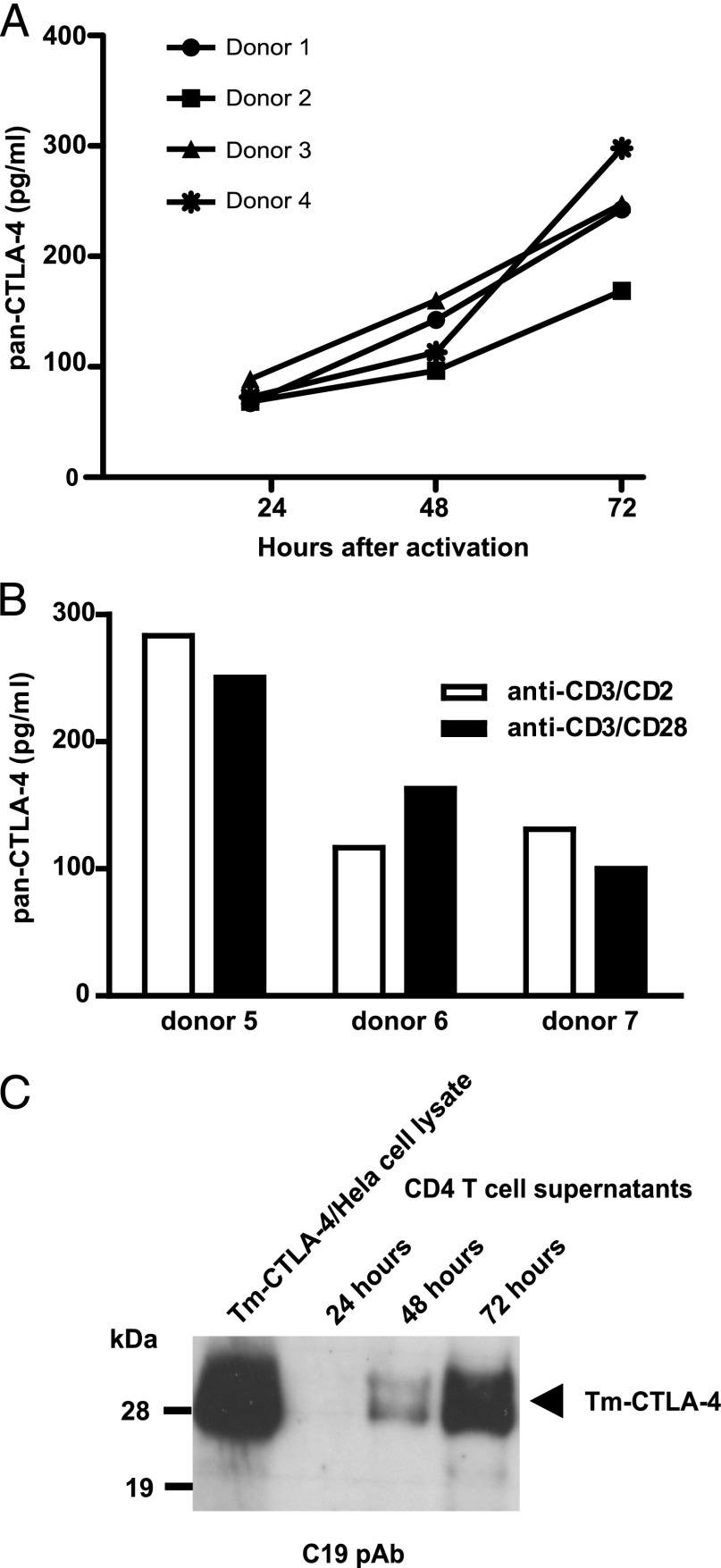
Immunoassay and Western blot analysis of Tm-CTLA-4 present in culture supernatants from activated CD4^+^ T cells. (**A**) Culture supernatants from 3 × 10^6^ cells/ml CD4^+^ T cells activated with anti-CD3/CD2 mAbs in vitro from four donors cultured on different days were harvested at the indicated time points and analyzed using the pan–CTLA-4 immunoassay. (**B**) Analyses of supernatants of CD4^+^ T cells (1 × 10^6^ cells/ml) of three donors cultured on different days stimulated in vitro for 72 h with either anti-CD3/CD2 mAbs or anti-CD3/CD28 microbeads. (**C**) Release of Tm-CTLA-4 in a time-dependent manner in culture supernatants of activated CD4^+^ T cells was confirmed by Western blot analysis (reducing gel) using Tm-CTLA-4 C terminus C19 polyclonal Ab. Western blot gel is representative of at least four independent experiments.

**FIGURE 5.**
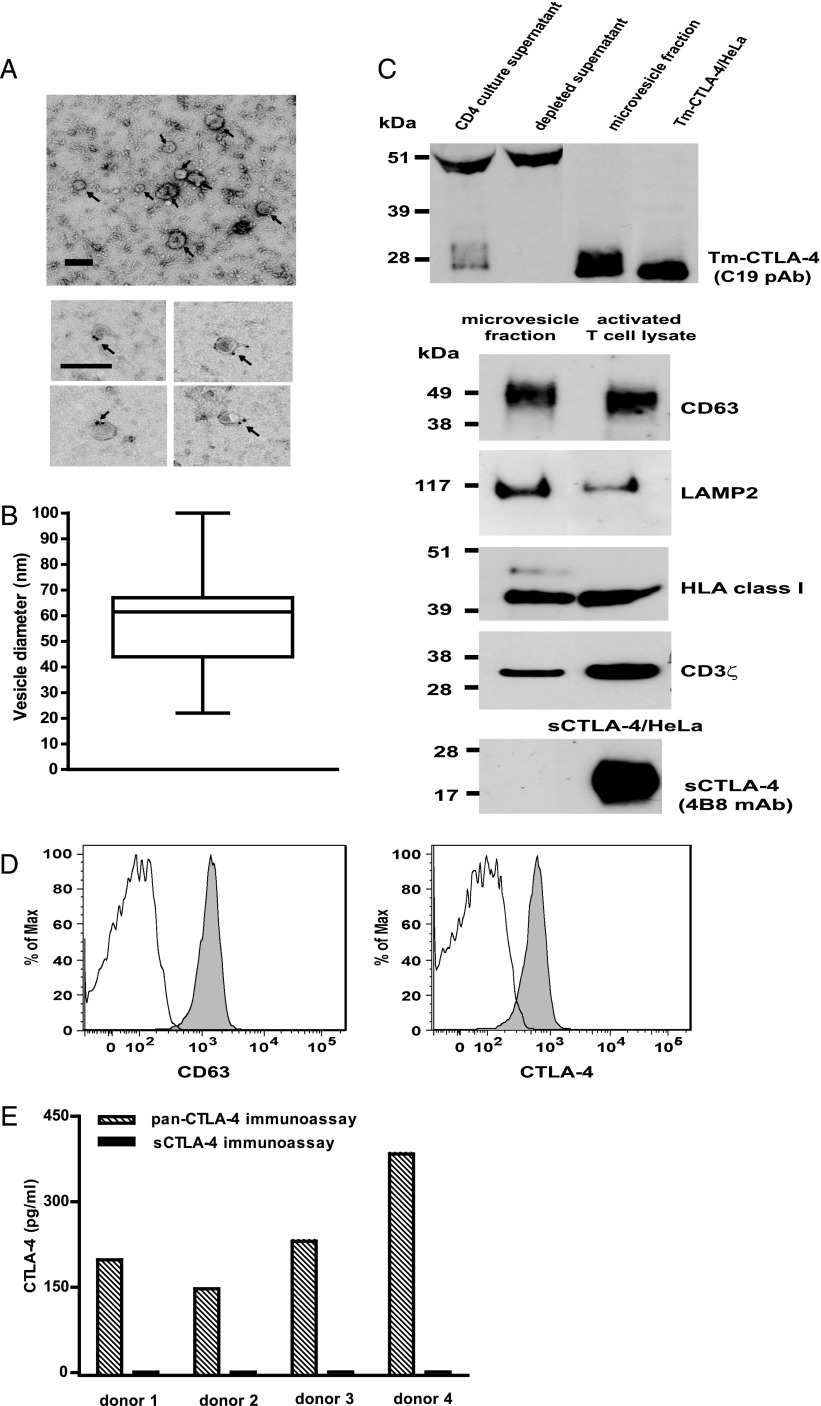
Characterization of exosome-like vesicles secreted by activated CD4^+^ T cells. (**A**) *Top panel*, Representative electron micrographs of microvesicles, as indicated by arrows, showing vesicles with low electron density as well as characteristic shape and size of exosomes (scale bar, 100 nm). *Bottom panel*, Anti-CD63 immunogold labeling of CD4^+^ T cell–derived microvesicles. Arrows indicate immunogold-positive labeling (scale bar, 100 nm). (**B**) The size distribution of microvesicles is plotted as a box plot; the box spans the 25th and 75th percentile, the horizontal line represents the median diameter, and the whiskers mark the extremes of the distribution (*n* = 24 microvesicles). (**C**) *Top panel*, Western blot analysis (reducing gel) of activated CD4^+^ T cell supernatant before and after ultracentrifugation, microvesicle fraction isolated from the T cell supernatant, and detergent lysates from Tm-CTLA-4/HeLa cells with anti–Tm-CTLA-4 C19 polyclonal Ab; *bottom panels*, Western blot analysis of microvesicle fraction isolated from the T cell supernatant with anti-CD63, anti-HLA class I, anti-CD3ζ (nonreducing gels), anti-LAMP2, and anti–sCTLA-4 Abs (reducing gels). CD4^+^ T cell or sCTLA-4/HeLa detergent lysates were used as positive controls. (**D**) Microvesicle fraction derived from activated CD4^+^ T cells was coated onto latex beads, surface stained with anti-CD63 and anti-CTLA-4 (BN13) (filled gray histogram) Abs or the corresponding isotype controls (open histogram), and analyzed by flow cytometry. (**E**) sCTLA-4 is not expressed in exosomal-like vesicles. The microvesicle fractions were isolated from supernatants of activated CD4^+^ T cells of four donors after 3 d of stimulation with anti-CD3/CD2 mAb and then tested in the pan–CTLA-4 and sCTLA-4 immunoassays. All experiments are representative of at least three independent observations.

Remarkably, we observed that, in the pan–CTLA-4 immunoassay (Fig. 4A, 4B), in which both CTLA-4 isoforms can be detected, the CTLA-4 concentration in CD4^+^ T cell supernatant samples was ∼10-fold higher than that observed using the sCTLA-4 immunoassay with either anti-CD3/CD2 mAb or anti-CD3/CD28 microbeads (compare Fig. 4A, 4B and Fig. 3A, 3B). CD4^+^ T cell supernatants were therefore analyzed by Western blot for the presence of Tm-CTLA-4. A band corresponding to Tm-CTLA-4 was detected in 48- and 72-h activated T cell supernatants (Fig. 4C). Because the immunoblot was probed with an Ab against the intracellular domain of Tm-CTLA-4 and the apparent molecular mass of the band corresponded to the entire Tm-CTLA-4 protein, proteolytic cleavage of the transmembrane receptor from the T cell surface does not account for the release of Tm-CTLA-4. We therefore tested the hypothesis that Tm-CTLA-4 is expressed in the microvesicles previously described to be secreted from activated T cells (30). T cell supernatants were sequentially ultracentrifuged to isolate the microvesicle-containing fraction. Electron-microscopic analysis of the pellet showed membrane-encapsulated vesicles with a size ranging between 20 and 100 nm, with cup-shaped morphology similar to that reported for cell-derived vesicles of endocytic origin termed exosomes (Fig. 5A, 5B). Moreover, these microvesicles express CD63, a tetraspanin shown to be enriched in exosomes (Fig. 5A, *bottom panel*) (31). Western blot analysis further showed that Tm-CTLA-4, CD63, LAMP2 (another established constituent of exosomes), HLA class I, and T cell type-specific protein CD3ζ were detected in the vesicle-containing fraction purified from supernatants of activated CD4^+^ T cells (Fig. 5C) (27, 31). No sCTLA-4 was observed in the ultracentrifuged pellet (Fig. 5C, *lowest panel*). We confirmed expression of surface CD63 and CTLA-4 on CD4^+^ T cell–derived exosomal-like vesicles by flow cytometry (Fig. 5D). Furthermore, exosome-enriched fractions isolated from culture supernatants of 72-h in vitro activated CD4^+^ T cells from four healthy donors were analyzed using the two CTLA-4–based assays, confirming the presence of mvCTLA-4, but not sCTLA-4 (Fig. 5E).

### Analysis of circulating sCTLA-4 in human serum

**FIGURE 6.**
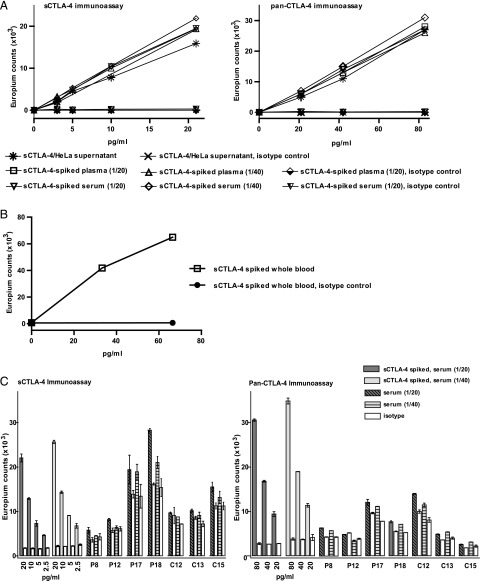
Detection of sCTLA-4 protein in plasma and serum samples. (**A**) Analysis by sCTLA-4 (*left panel*) and pan–CTLA-4 (*right panel*) immunoassays of 2-fold serial dilutions of recombinant sCTLA-4 spiked into 1/20 and 1/40 dilutions of plasma and serum samples. An isotype-matched mouse IgG2a aκ capture Ab is used as a negative control. Serial dilutions of culture supernatant of sCTLA-4 HeLa cell transfectants in standard assay buffer were included in each assay. (**B**) Detection of recombinant sCTLA-4 spiked into human whole blood. Mouse IgG2a aκ isotype-matched Ab used as capturing reagent served as a negative control. (**C**) Analysis of sCTLA-4 in serum samples from autoimmune patients and controls using the sCTLA-4 immunoassay (*left panel*) and the pan–CTLA-4 immunoassay (*right panel*). Serial dilutions of recombinant sCTLA-4 were spiked into serum diluted 1:20 (dark gray columns) and 1:40 (light gray columns), and representative examples from a total of 61 donors are shown (see Supplemental Fig. 3 for results from all donors), as follows: 4 GD patients and 3 age- and sex-matched, healthy volunteers, sera diluted 1/20 (dark shaded columns) and 1/40 (light shaded columns). For all samples, including the spiked serum samples, the mouse IgG2aκ isotype control is shown (open columns). Columns, mean of duplicate measurements; bars, ±SD.

Previous studies, using Ab-based assays in which both capture and detection Abs were specific for the N-terminal region of CTLA-4, reported circulating levels of sCTLA-4 in serum from patients with autoimmune diseases in the 2–97 ng/ml range (18–25). To investigate whether sCTLA-4 could be detected in serum or plasma samples using a sCTLA-4–specific immunoassay, we first determined plasma and serum tolerability in both assay formats (sCTLA-4 specific and pan–CTLA-4) and found that a 1/20 dilution was the highest concentration of both biological fluids that could be tested without observing >20% inhibition of samples spikes with sCTLA-4 protein (data not shown). Thus, based on the spike and recovery analysis in 1/20 dilutions of serum and plasma, the lower limit of detection of recombinant sCTLA-4 in neat serum and plasma is 40 pg/ml in the sCTLA-4– specific assay and 400 pg/ml in the pan–CTLA-4 assay. Spike and recovery experiments were also performed as represented in Fig. 6A using 1/20 and 1/40 sera and plasma dilutions spiked with 2-fold serial dilutions of recombinant sCTLA-4. In both assays, we obtained good recovery and dilutional linearity (all r^2^ > 0.98) of spiked sCTLA-4 with sensitivity and detection limits similar to those measured in the culture medium of sCTLA-4/HeLa transfectants (Fig. 6A, Supplemental Fig. 2A, 2C, *right panels*). Good spike recovery was also observed in plasma samples derived from heparinized whole human blood that had been incubated with recombinant sCTLA-4 at 37°C for 2 h, thus providing evidence that sCTLA-4 is likely to be stable in the circulation in vivo (Fig. 6B).

We then examined serum at 1/20 and 1/40 dilutions from individuals affected with T1D and GD compared with age- and sex-matched healthy controls using the sCTLA-4 as well as the pan–CTLA-4 assay (Fig. 6C for representative data; Supplemental Fig. 3A–D shows raw data for all samples). In both assay formats, the binding determined with the anti–CTLA-4 capture Ab was compared with an isotype-matched control to detect the presence of heterophilic Abs or other serum components that bind mouse Ig molecules; such molecules interfere with the interpretation of immunoassays (32, 33) and are present in varying levels in human plasma and serum. The definition for positive samples was a signal above the isotype control background that proportionally decreased as a consequence of serum dilution, as exemplified by the spiked serum samples (1/20 and 1/40 dilutions) on the left-hand portion of each panel of Fig. 6C, in which the specific signal decreased with dilution, whereas the signal observed from the isotype control remained the same. In Fig. 6C, seven representative samples from the GD patients and matched healthy control cohorts (Supplemental Fig. 3A, 3B) are shown for both assays at 1/20 and 1/40 dilutions. In all the patient and control serum samples, except for GD P18, similar signal intensities were obtained using 20-fold and 40-fold serum dilutions in both the specific and isotype control assay in both formats (Fig. 6C, Supplemental Fig. 3). Interestingly, in the sCTLA-4 immunoassay, GD P18 was the only sample for which we observed ∼2-fold signal reduction at the 1/40 serum dilution compared with the 1/20 serum dilution, whereas the readout from the isotype control remained similar for both dilutions (Fig. 6C, *left panel*). The concentration of sCTLA-4 in GD P18 serum, determined using the sCTLA-4 immunoassay, is 260 pg/ml and is below the limit of detection of the N terminus pan–CTLA-4 immunoassay (Fig. 6C, *right panel*). Thus, the concentration of circulating sCTLA-4 that we have detected in this single patient is at least 10-fold lower than the 2,000–97,000 pg/ml sCTLA-4 that has been reported for autoimmune patients in previous studies (18–25). Overall, our results indicate that sCTLA-4 does not represent a significant serum protein component in autoimmune diseases.

On a technical note, the result of all sera tested in both assay formats (Supplemental Fig. 3) reinforces previous observations that heterophilic Abs (32–34) are present in variable levels in human serum and can be a confounder of Ab-based immunoassays. In Supplemental Fig. 3, we have organized the results in the CTLA-4 portion of the assay from lowest to highest in each cohort to demonstrate that the variation of binding by the samples in the specific assay is generally mimicked by the isotype binding, thus suggesting that a background anti-mouse IgG reactivity is being observed and that this varies between people. It is notable that the pan–CTLA-4 immunoassay has in general less background than the sCTLA-4 immunoassay. Possibly this is the consequence of the different anti–CTLA-4 detection reagents; the pan–CTLA-4 detection reagent is directly biotinylated, whereas the anti–sCTLA-4 reagent is detected by a secondary biotinylated anti-mouse IgG2b mAb.

### Tm-CTLA-4 associated with microvesicles is not detected in human serum

mvCTLA-4 was not detected at levels >400 pg/ml in serum of T1D and GD patients or matched healthy volunteers using the pan–CTLA-4 assay (Fig. 6C, *right panel*, and Supplemental Fig. 3B, 3C). We further investigated whether isolation of microvesicles from serum could increase the sensitivity of Tm-CTLA-4 detection. Microvesicles prepared from serial ultracentrifugation of sera from T1D and healthy volunteers were analyzed by Western blot and found to express CD63 and CD3ζ (Supplemental Fig. 4). As expected due to the diverse tissue sources of microvesicles in the blood (27), CD63, a constitutive marker of all exosomes (31), was significantly more abundant than the T cell–specific CD3ζ molecule as compared with the microvesicle-like exosomes that were isolated from activated CD4^+^ T cells (Supplemental Fig. 4, Fig. 5C). mvCTLA-4 was not found in the serum-derived microvesicles by Western blot analysis (data not shown), confirming the results of the pan–CTLA-4 immunoassay.

## Discussion

Biochemical and immunological characterization of the soluble isoform of CTLA-4 has been hindered by the lack of specific high-affinity reagents that bind to this isoform. In this study, we have developed and characterized several sCTLA-4–specific reagents, including two mAbs, and used them to measure sCTLA-4 levels present in vivo and from cultured primary, activated T cells. We have shown that, using a sCTLA-4 assay with a 2 pg/ml sensitivity in tissue culture supernatant samples, CD4^+^ T cells upon stimulation secrete 17–32 pg/ml sCTLA-4. Nonetheless, with a detection limit of 40 pg/ml in serum and plasma samples, sCTLA-4 was detected in only 1 of 32 serum samples from autoimmune disease patients and none of the samples from 29 healthy controls.

Our results are in contrast with previous studies reporting levels of circulating sCTLA-4 in the 2–97 ng/ml range in a large proportion of patients with various autoimmune diseases (18–25). The immunoassays used in these previous studies relied on Abs that recognize the IgV-like extracellular region of CTLA-4 (35), similar to the pan–CTLA-4 immunoassay employed in the current study; in only two is an isotype control used to adjust for individual variation in nonspecific binding, and none of the studies reported linearity of the binding assay used at the concentrations of serum tested by spiking with known concentrations of sCTLA-4 (18, 25). Recently, Tector et al. (26) showed that CTLA-4–positive plasma of patients with myasthenia gravis after purification and enrichment by an N terminus CTLA-4 mAb column specifically bound anti–CTLA-4 Abs and the CTLA-4 ligand CD80/CD86 fusion proteins. However, the eluted plasma material was also shown to be captured by a protein A column, unlike recombinant sCTLA-4, which was detected in the flow-through. This result indicates that the immunoreactive material isolated from the plasma, which had been captured by anti–CTLA-4 Abs and the CD80/CD86 molecules, both consisting of two extracellular Ig-like domains (36, 37) and fused to the Fc region of the mouse Ig, was not sCTLA-4, but a material that possessed Ig-like properties. Together with these recent findings from Tector et al. (26), our current results suggest that a reassessment of what are termed circulating levels of the sCTLA-4 isoform should be made and that the molecular basis of the CTLA-4–like immunoreactive material present in the circulation of some autoimmune patients and healthy controls warrants further study. However, even though circulating levels of the sCTLA-4 isoform are generally below the limit of sensitivity of our sCTLA-4–specific assay (40 pg/ml), this does not rule out the possibility that secreted sCTLA-4 plays a functional role locally in tissues in which activated T cells and APCs are interacting.

As predicted by its lack of the cysteine residue at position 122 (C122), which mediates the covalent homodimerization of CTLA-4 (38), our data indicate that recombinant sCTLA-4 from HeLa-transfected cells migrates as a monomer under nonreducing conditions. However, it has been reported that a mutation, which abrogates intermolecular disulfide bonding at C122, reduces but does not inhibit dimerization of CTLA-4. N-glycosylation at the Asn^113^ and Asn^145^ residues of the extracellular domain appears to promote the cys-independent dimerization of CTLA-4 (9). By treating sCTLA-4/HeLa transfectants with tunicamycin, an inhibitor of N-glycan processing, we have demonstrated that sCTLA-4 is N-glycosylated, and therefore the potential for cys-independent homodimer formation exists for secreted sCTLA-4. It is also possible that functional dimerization occurs after binding to CD80/CD86 ligands at the T cell–APC interface (9). Notably, we have also shown that sCTLA-4 secreted by primary human T cells has the same apparent molecular mass as fully N-glycosylated recombinant sCTLA-4. Furthermore, our data show a positive correlation between the low expression levels of sCTLA-4 protein detected in human T cells and the reported moderate increase of sCTLA-4 mRNA following in vitro activation (7, 11).

It is noteworthy that two of the anti-human sCTLA-4–specific Abs, 10D1 mAb and 4017 pAb, recognize mouse recombinant sCTLA-4. We have shown in transfected HeLa cells that, like human sCTLA-4, mouse sCTLA-4 is secreted as an N-glycosylated monomer. Of interest, given their higher molecular masses, both human and mouse sCTLA-4 secreted into the culture media might represent the fully glycosylated form compared with the protein detected in the cell lysates. Furthermore, variation in N-glycosylation based on protein structure probably accounts for the molecular mass differences of the mouse Tm-CTLA-4 and sCTLA-4 proteins relative to their human counterparts.

The biological relevance of our observations requires further investigation. Despite being expressed as a monomer, recombinant sCTLA-4 was reported to block T cell proliferation, an effect that could be dampened by the addition of anti–CTLA-4 Abs (7). Recombinant sCTLA-4 was also able to inhibit the costimulatory signal of Chinese hamster ovary cells expressing CD80 similarly to what was observed after addition of CTLA-4Ig (7). sCTLA-4Ig has been shown to modulate the CD28/CD80-CD86 pathway and inhibit T cell activation (39–44); in clinical trials, formulations of CTLA-4Ig have proved to be effective in the treatment of autoimmune diseases and allograft rejection (45–51). Thus, sCTLA-4, in vivo, might modulate local T cell–APC interaction with a mode of action similar to the one observed for the CTLA-4Ig. Although, in vivo, the secretion of sCTLA-4 might represent a further mechanism to dampen an ongoing immune response, the release of this molecule might also be a distinctive feature of a specialized subset of T cells such as Tregs that constitutively express and require CTLA-4 for their suppressive activity (5, 6, 52).

Another major finding of our study is that microvesicles that express covalent homodimers of Tm-CTLA-4, which we have termed mvCTLA-4, are secreted in the culture supernatant of activated CD4^+^ T cells. The low electron density regular cup-shaped microvesicles isolated from the culture supernatants with a diameter between 20 and 100 nm and expressing CD63 and LAMP2 (28, 53) presented characteristics typical of exosomes, small membrane vesicles of endocytic origin that have been shown to carry a specific array of proteins, mRNA and miRNA (31, 54). Exosomes have been shown to be secreted by both innate and adaptive cells (30, 53, 55, 56) in various biological fluids such as blood, saliva, urine, and breast milk (27, 57–59), where they appear to play an important role in intercellular communication and immunoregulation (30, 53, 55, 60). It has been reported that CD4^+^ T cells activated via the TCR secrete vesicles that exhibit characteristics and markers of exosomes and express T cell adhesion molecules, such as CD2 and CXCR4, and the TCR/CD3/ζ complex (30, 61). Others have shown that activated T cells release exosomes expressing apoptosis-inducing molecules such as FAS ligand and APO2 ligand (62). Exosomes from activated T cells have been shown to endow APCs with the capacity to stimulate or inhibit T cell responses (63, 64) or directly induce proliferation in autologous resting T cells accompanied by a change in the cytokine/chemokine profile (65). We speculate that the presence of Tm-CTLA-4 on microvesicles/exosomes might be responsible for a portion of the biologic functions ascribed in these published studies, and we are currently investigating the ability of mvCTLA-4 to inhibit CD80/CD86-mediated costimulation.

Of note, mvCTLA-4 was not detected, using the pan–CTLA-4 immunoassay, in human sera of GD and T1D patients or healthy controls, even after isolation of microvesicles. Nevertheless, we cannot exclude that the lack of detection of microvesicles carrying Tm-CTLA-4 was due to the large proportion of non-T cell–derived microvesicles in the serum samples, and mv-CTLA-4 might be detectable in serum from humans having a high degree of cell activation such as in acute graft-versus-host disease.

We suggest that these two forms of CTLA-4, sCTLA-4 and mvCTLA-4, constitute novel mechanisms by which T cells can alter in the local milieu APC function via binding to CD80/CD86 ligands. Release of sCTLA-4 forms possibly by directional secretion as shown for IL-2 and IFN-γ (66) from a T cell subset such as activated Tregs might modify expression of CD80 and CD86 on nearby APCs, thereby influencing CD28 engagement (6, 67, 68), as well as inducing immunosuppression via, for example, an IDO-dependent pathway (69). In conclusion, our study provides novel evidence documenting the secretion of both sCTLA-4 and mv-CTLA-4 from human primary, activated T cells and prompts further investigation to gain insight into their functions.

## Supplementary Material

Data Supplement
