# Supplementary material for: Investigation of Soluble and Transmembrane CTLA-4 Isoforms in Serum and Microvesicles
Source: J Immunol. 2014 Jun 13;193(2):889–900. doi: 10.4049/jimmunol.1303389 (PMC4082723; doi:10.4049/jimmunol.1303389)
Supplement: Data Supplement [file supp_193_2_889__index.html]

Investigation of Soluble and Transmembrane CTLA-4 Isoforms in Serum and Microvesicles — Data Supplement 

# Investigation of Soluble and Transmembrane CTLA-4 Isoforms in Serum and Microvesicles

## Data Supplement

**Files in this Data Supplement:**

- Supplemental Figures 1 (PDF)
