# Supplementary material for: Investigation of Soluble and Transmembrane CTLA-4 Isoforms in Serum and Microvesicles
Source: J Immunol. 2014 Jun 13;193(2):889–900. doi: 10.4049/jimmunol.1303389 (PMC4082723; doi:10.4049/jimmunol.1303389)
Supplement: Data Supplement [file 1303389_JI_1303389_Supplemental_Figures_1.pdf]

Supplemental Figure 1

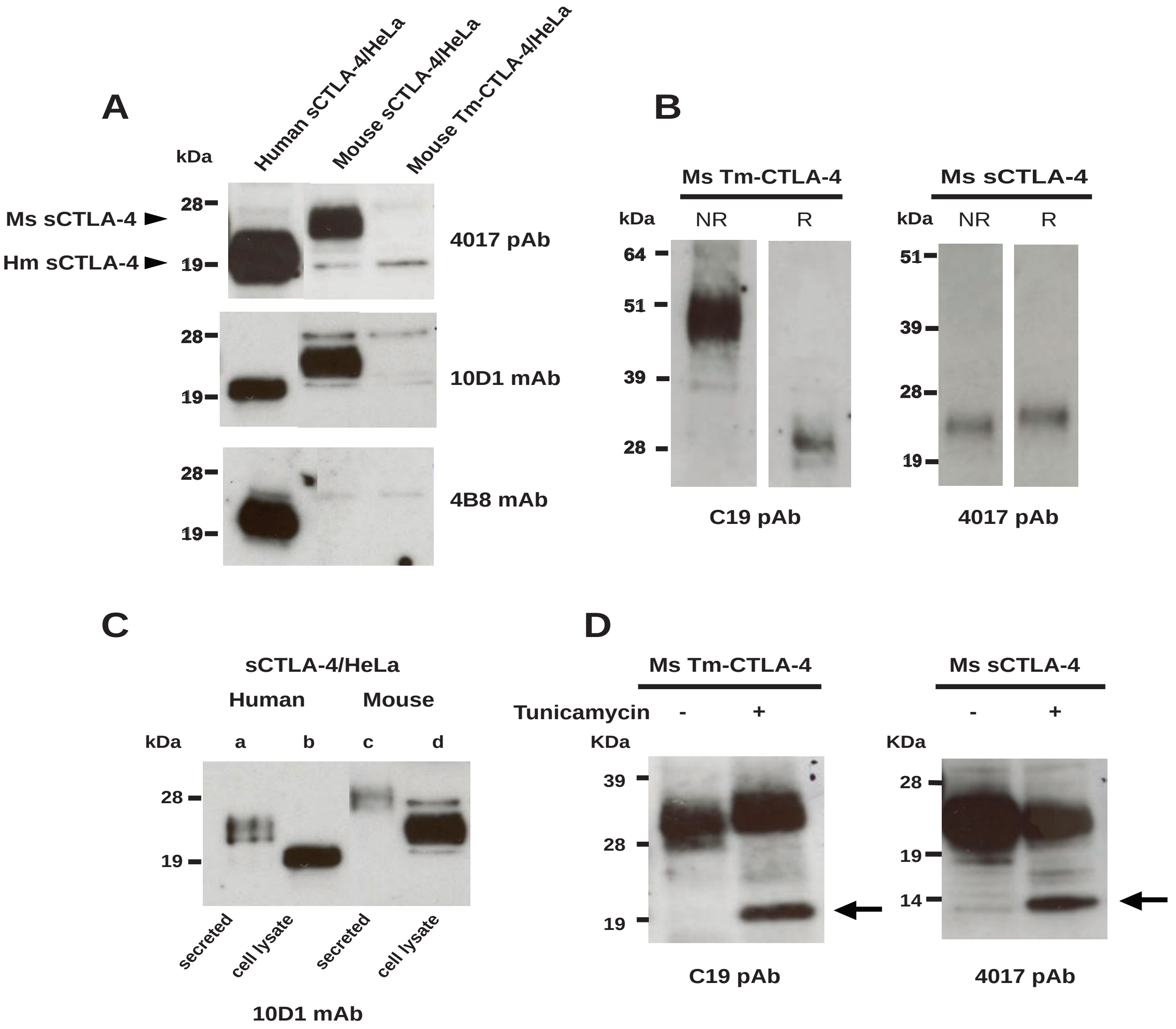

Supplemental Figure 1. Biochemical characterization of mouse Tm-CTLA-4 and sCTLA-4. (A) Detergent lysates of HeLa cells expressing recombinant human sCTLA-4, mouse sCTLA-4 and mouse Tm-CTLA-4 were subjected to Western blot analysis under reducing conditions with the indicated anti-human sCTLA-4 antibodies. 4017 polyclonal Ab and 10D1 mAb but not 4B8 mAb specifically recognize the mouse CTLA-4 soluble isoform. (B) Mouse Tm-CTLA-4 migrates as a dimer under non-reducing conditions (NR) and as a monomer under reducing conditions (R). Mouse sCTLA-4 is expressed as a monomeric protein under non-reducing and reducing conditions. (C) Western blot analysis under reducing condition using 10D1 mAb to probe detergent lysates and culture supernatants derived from HeLa transfectants expressing human (lanes a and b) and mouse sCTLA-4 (lanes c and d). (D) HeLa cells expressing mouse Tm-CTLA-4 or sCTLA-4 recombinant proteins were treated with tunycamicin prior to Western blot analysis under reducing conditions with C19 and 4017 polyclonal Ab. Arrows indicate unglycosylated forms of Tm-CTLA-4 and sCTLA-4 migrating at their predicted molecular masses. Western blot gels are representative of at least three independent experiments.

Supplemental Figure 2

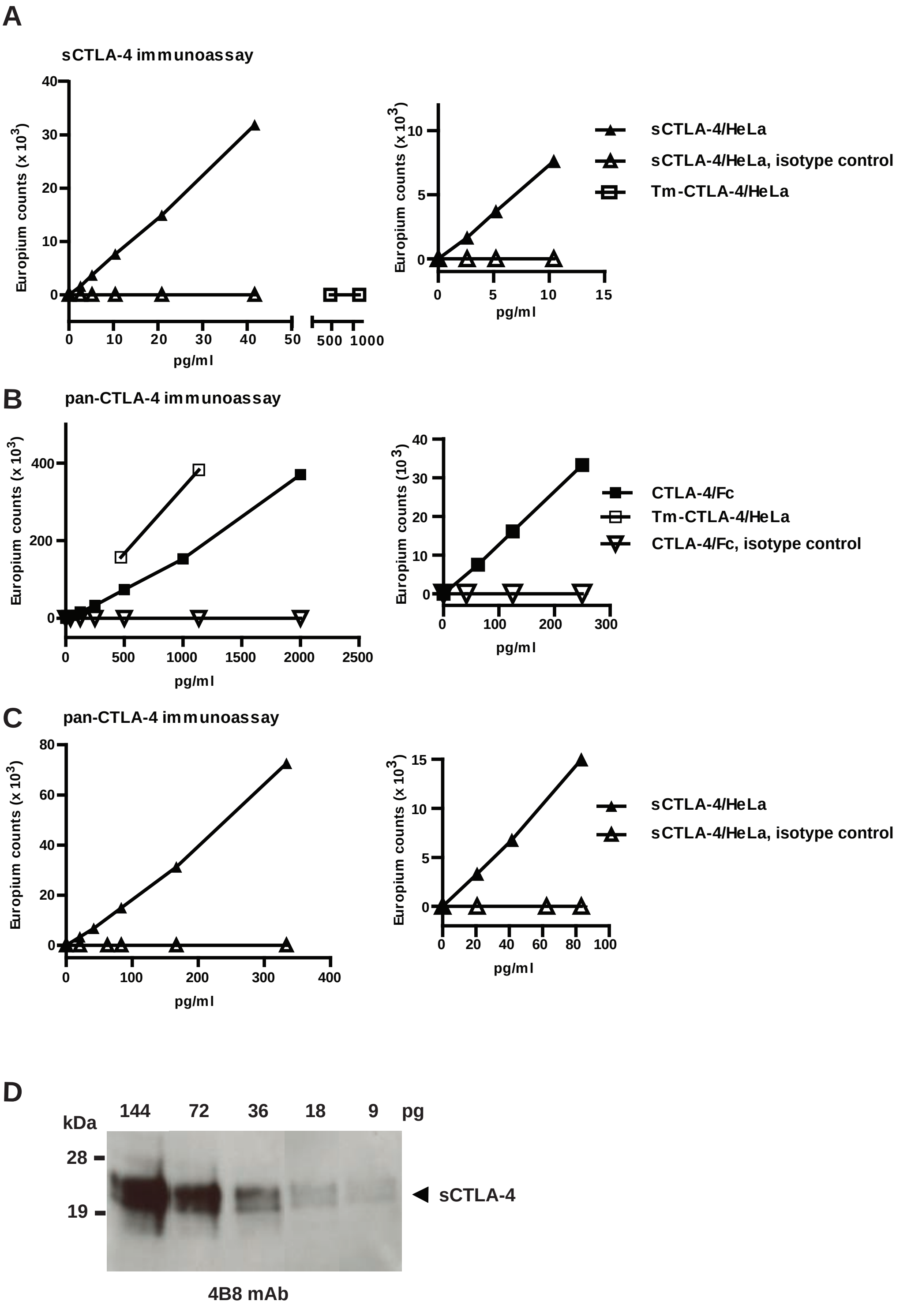

Supplemental Figure 2. Human sCTLA-4 immunoassay calibration curves. (A) Linear detection is observed for 2-fold serial dilutions of recombinant sCTLA-4 secreted into the culture medium of HeLa transfectants (sCTLA-4/HeLa) as measured by the sCTLA-4 immunoassay (closed triangles). Tm-CTLA-4 protein from transfected HeLa cell-derived detergent lysates (1 x 10^4 and 5 x 10^3 cells) is not detected in this assay format (open squares); recombinant secreted sCTLA-4 protein did not bind to an isotype matched $IgG_{2a\kappa}$ antibody (open triangles). In data not shown, binding of the CTLA-4/Fc fusion protein was also not detected. (B, C) Quantitation of secreted recombinant sCTLA-4 calculated in a pan-CTLA-4 immunoassay (capture: BN13 mAb, detection: biotinylated 14D3 mAb) using serial dilutions of CTLA-4/Fc fusion protein for the standard curve. Detergent lysates from Tm-CTLA-4/HeLa cells (1 x 10^4 and 5 x 10^3 cells) served as positive controls. Isotype-matched $IgG_{2a\kappa}$ antibody as the capture reagent was used as a control for non-specific binding, such as observed with heterophilic antibodies. (D) Limit of detection of sCTLA-4 protein secreted into culture supernatant of HeLa cell transfectants analyzed by Western blot. Serial dilutions of secreted sCTLA-4 starting at 144 pg per lane were analyzed by Western blot using 4B8 mAb. The lower limit of sCTLA-4 protein detected by Western blot is approximately 9 pg per lane (equating to loading a maximum of 37 microliters per lane of a 243 pg/ml solution). All experiments are representative of at least three independent observations.

Supplemental Figure 3

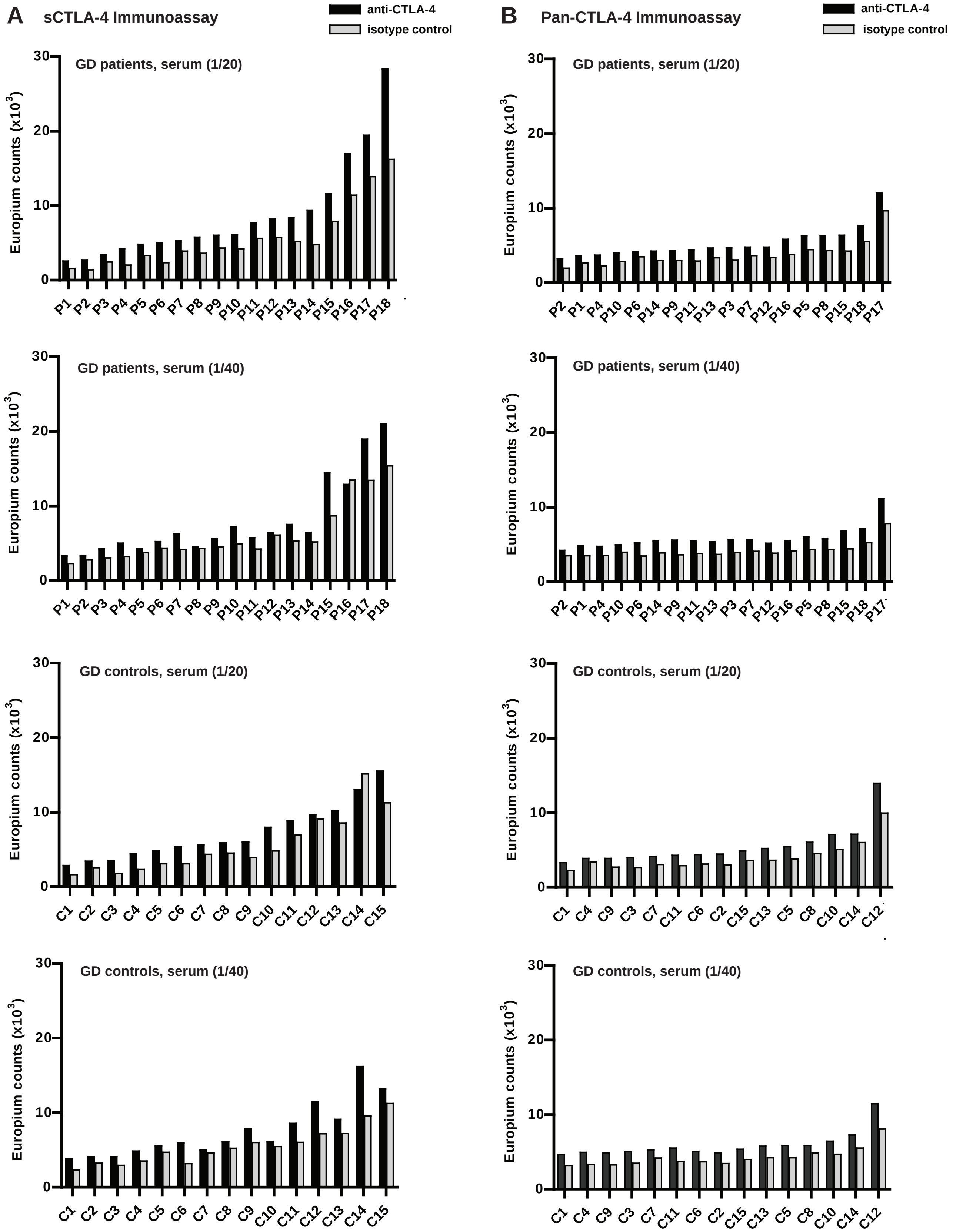

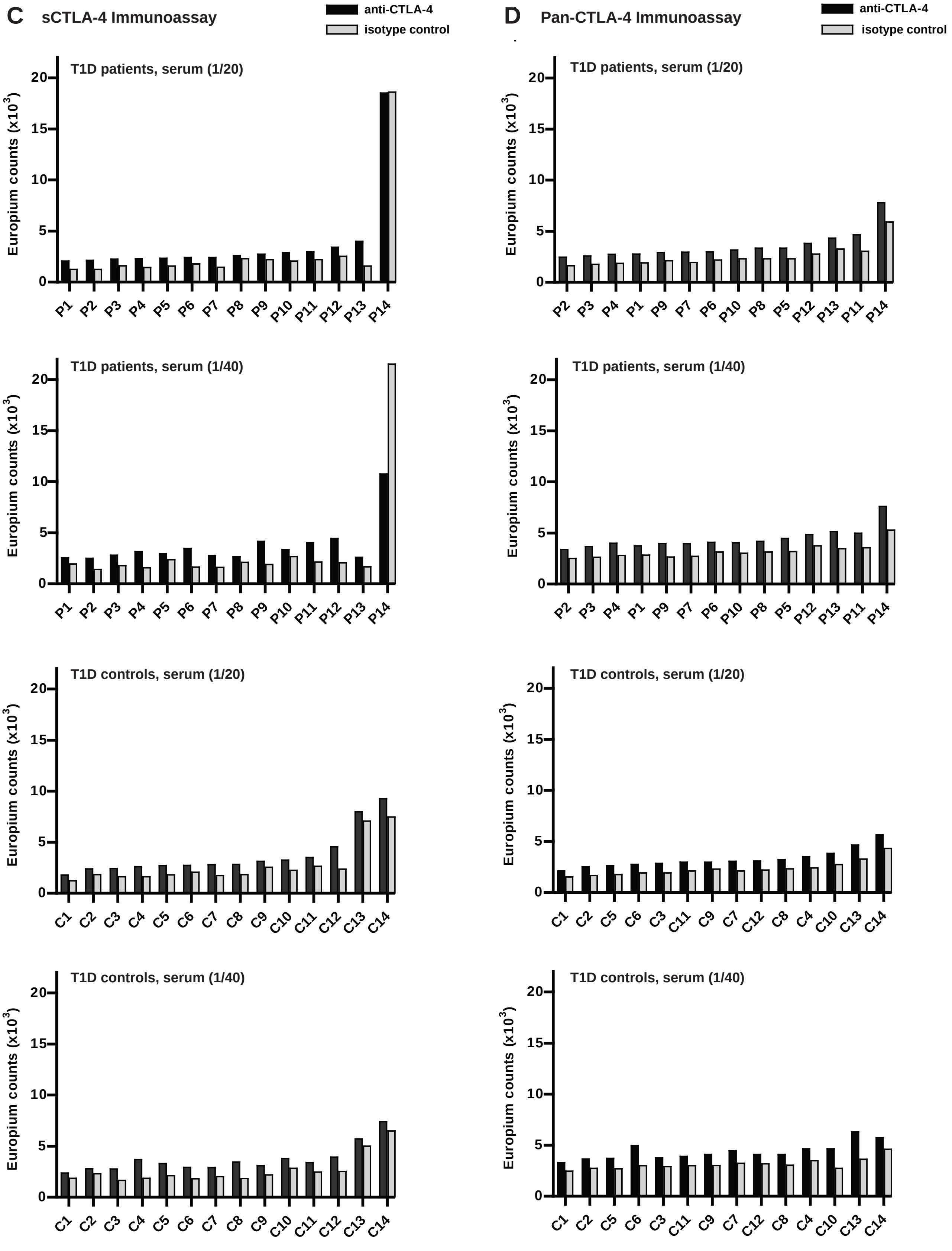

Supplemental Figure 3. Assessment of sCTLA-4 in serum samples from GD and T1D patients and control cohorts. Serum samples from 18 GD patients and 15 age- and sex-matched healthy controls (A, B) and 14 T1D patients and 14 age- and sex-matched healthy controls (C, D) diluted 1/20 and 1/40 were tested with the sCTLA-4-specific immunoassay and the pan-CTLA-4 immunoassay (filled columns). Each sample was also tested for binding to an isotype-matched mouse $IgG_{2a\kappa}$ capture antibody (open columns). Columns are the mean of duplicate measurements. Samples in each of the disease and control cohorts are labelled (P1 through P18, for example in Supplemental 3A) based on their ranking from the lowest to highest europium counts in the sCTLA-4 assay as tested at a 1/20 dilution (Supplemental 3A and 3C, first and third graphs) to facilitate comparison with the pan-CTLA-4 assay results where the lowest to highest europium counts are also presented.

Supplemental Figure 4

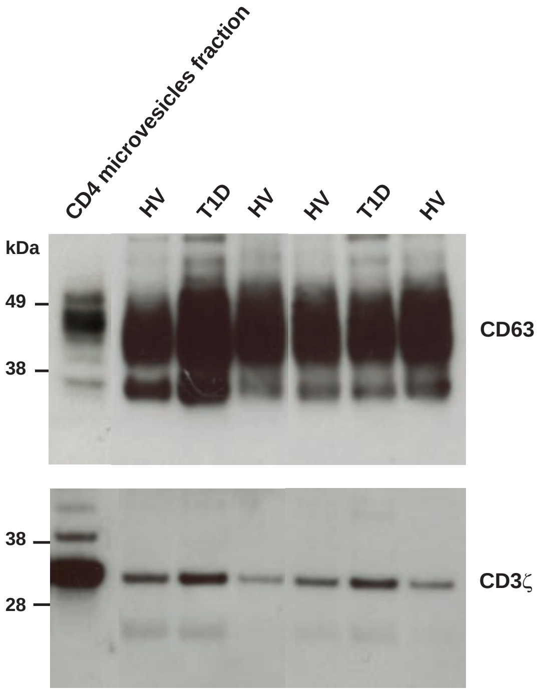

Supplemental Figure 4. Analysis of serum-derived microvesicles. Microvesicles were isolated from sera of T1D patients and healthy volunteers (HV) by sequential ultracentrifugation and analyzed by Western blot using anti-CD63 and anti-CD3ζ mAbs. Western blot gel is representative of at least four independent experiments.
